# Supplementary material for: Probiotic and Triticale Silage Fermentation Potential of Pediococcus pentosaceus and Lactobacillus brevis and Their Impacts on Pathogenic Bacteria
Source: Microorganisms. 2019 Sep 4;7(9):318. doi: 10.3390/microorganisms7090318 (PMC6780645; doi:10.3390/microorganisms7090318)
Supplement: Supplementary file 1 [file microorganisms-07-00318-s001.pdf]

microorganisms

MDPI

Probiotic and *Triticale* Silage Fermentation potential of *Pediococcus pentosaceus* and *Lactobacillus brevis* and Their Impacts on pathogenic bacteria

Ilavenil Soundharrajan [a]†, Da Hye Kim[2]†, Palaniselvam Kuppusamy [a], Karanan kuppusamy[a]

Hyun Jeong Lee [c], Ki Choon Choi [a*]

[a] Grassland and Forage division, National Institute of Animal Science, Rural Development Administration, Cheonan, South Korea-330-801.

[b]Center for Research on Environmental Disease, College of Medicine, University of Kentucky, 1095 VA Drive, Lexington, KY 40536 USA.

[c]Jangsu Agriculture Technology Center, Jangsu, Jeollabuk-do, 55640, South Korea

†They are contributed equally to this work

[*] **Corresponding author.** Grassland and Forage division, National Institute of Animal Science, Rural Development Administration, Cheonan, South Korea-330-801. Tel.: +82-41-580-6752, Fax: +82-41-580-6779.

E-mail address: [*]choiwh@korea.kr [K.C. Choi]

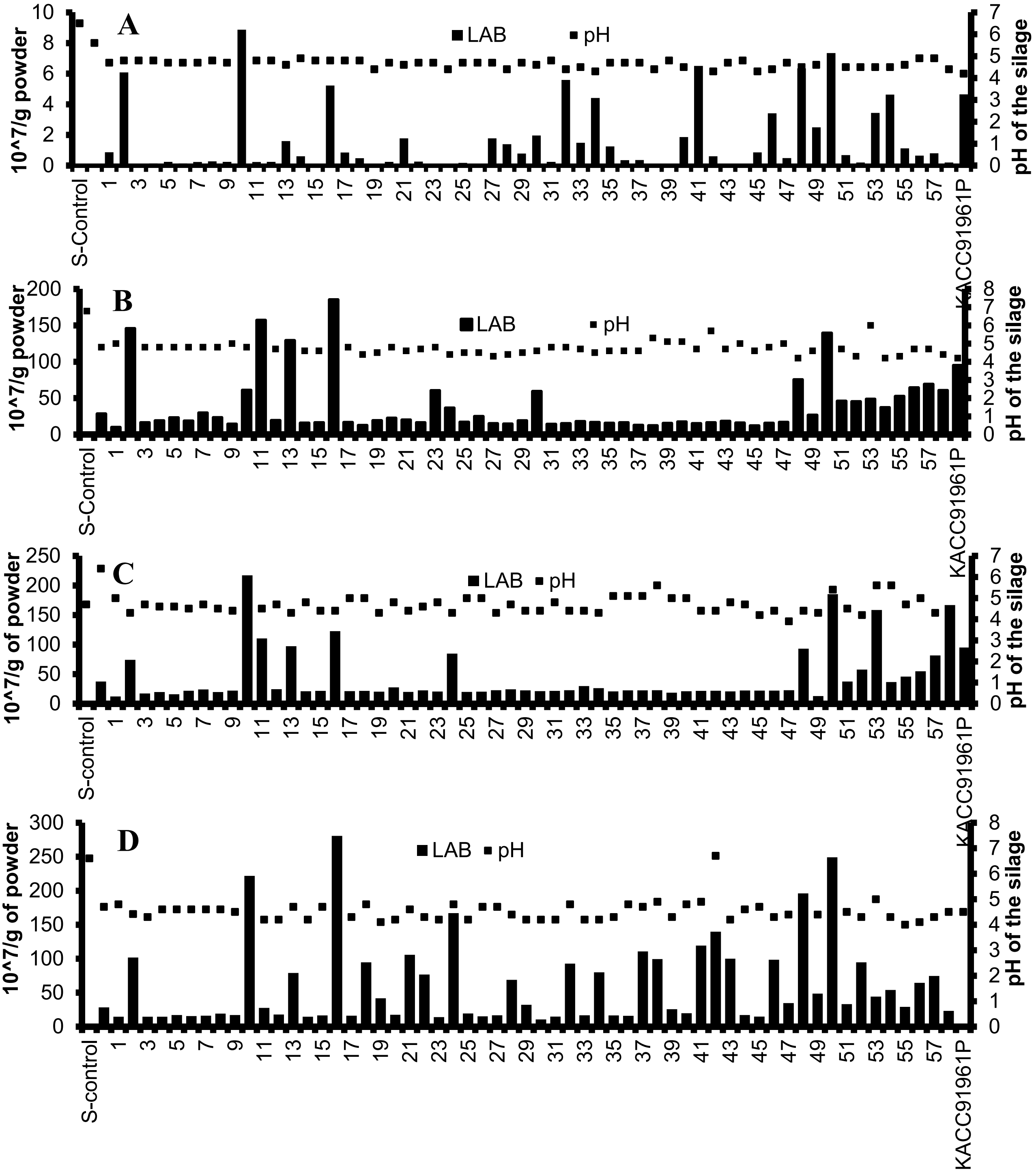

Supplementary figure S1: Effect of lactic acid bacteria on the fermentation of triticale silage. (A) microbial population and pH of the silage at 40% moisture; (B) microbial population and pH of the silage at 50% moisture; (C) microbial population and pH of the silage at 60% moisture; (D) microbial population and pH of the silage at 70% moisture**.** S- Control (Sterilized with Ethylene Oxide gas), Control – Without addition of LAB. LAB- lactic acid bacteria.
